# The Analysis of Leaf Traits of Eight *Ottelia* Populations and Their Potential Ecosystem Functions in Karst Freshwaters in China

**DOI:** 10.3389/fpls.2018.01938

**Published:** 2019-01-07

**Authors:** Yu Cao, Yang Liu, Leah Ndirangu, Wei Li, Ling Xian, Hong Sheng Jiang

**Affiliations:** ^1^Key Laboratory of Aquatic Botany and Watershed Ecology, Wuhan Botanical Garden, Chinese Academy of Sciences, Wuhan, China; ^2^Hubei Key Laboratory of Wetland Evolution and Ecological Restoration, Wuhan Botanical Garden, Chinese Academy of Sciences, Wuhan, China; ^3^University of Chinese Academy of Sciences, Beijing, China; ^4^Sino-African Joint Research Center, Chinese Academy of Sciences, Wuhan, China

**Keywords:** *Ottelia*, Crassulacean acid metabolism, bicarbonate usage, leaf traits, shallow freshwaters

## Abstract

Submerged macrophytes play a structuring role in the shallow freshwater ecosystem by increasing the heterogeneous state in freshwaters. The macrophytes in genus *Ottelia* were featured for their broad leaves, which might consequently produce specialized functions that differed from other submerged species. To explore the potential ecological role of *Ottelia*, a field investigation was conducted on leaf traits in eight populations of *Ottelia* ranging from the southwestern Yunnan–Guizhou plateau to the southern Hainan island in China covering a distance of >1,700 km. The eight populations included all the extant *Ottelia* species and varieties in China except the well-documented *O. alismoides*. Carbon-related traits [bicarbonate usage, photosynthetic characteristics, capability of Crassulacean acid metabolism (CAM)], pigment content and parameters of chlorophyll fluorescence, morphology and mass of the leaves were determined. The different populations showed distinct functional traits of mature leaves; *O. acuminata* var. *songmingensis* had the thickest and longest leaf with CaCO_3_ precipitation on the both sides of the leaf, and *O. cordata* showed putative CAM activity with the highest diel acidity changes 12.5 μequiv g^-1^ FW. Our results indicated an important role of *Ottelia* populations in carbon cycling as the dominant species in karst freshwaters in China.

## Introduction

Submerged macrophytes are considered as one of the most important primary producers in shallow oligotrophic freshwaters and strongly affect the nutrient turnover for freshwater ecosystem (Wetzel, 1964; Epstein et al., 2012; Olsen et al., 2017). In addition, submerged macrophytes can interact with other organisms, e.g., protecting the zooplankton from fish grazing or providing substrate for periphyton growth (Jeppesen et al., 1998; Cao et al., 2014, 2017). Plant functional traits, as a series of core properties describing the growth, survival and reproduction of plants, are useful tools to explore the ecological function of submerged macrophytes in freshwater systems (Grime, 1974). Most studies related with plant functional traits are focusing on terrestrial forest or grass (Kraft et al., 2008; Klimešová et al., 2016). For example, Kraft et al. (2015) stated that using the combination of functional traits (e.g., the growth form), but not simplistic usage of single functional trait, was important to infer community assembly processes in grassland. For submerged macrophytes, there are only few recent studies related with functional traits, which have investigated the functional traits at the community level along water depth gradients in natural lakes (Fu et al., 2014, 2018; Liu and Wang, 2018).

Leaf is the most important photosynthetic organ, and leaf traits are one of central plant functional traits (Petter et al., 2016; Damián et al., 2018). Especially the concept of ‘leaf economic spectrum’ (LES) has revealed the importance of leaf traits; a typical trade-off between leaf functional traits of >2,000 species has been found, which shows that leaves with the higher photosynthesis rate are featured with shorter life span and lower leaf mass per area (MPA; Wright et al., 2004). In addition, Cornwell et al. (2010) linked the decomposition rate of terrestrial plant litter with the position of the species in the LES, indicating a close relationship between leaf traits and ecosystem function. The submerged macrophytes also have contrasting decomposition rates (*Potamogeton crispus* vs. *P. macckianus*), which can significantly affect the carbon cycling in shallow lakes (Wang et al., 2016). However, none of submerged species has been included in LES. Compared with other submerged species, leaves of *Ottelia* are usually much broader with long petiole, and meantime these leaves can play an extra role in ecosystem carbon cycling through CaCO_3_ precipitation on the leaf surface compared with those of terrestrial plants (Prins et al., 1982; Yin et al., 2017).

The genus *Ottelia* widely distributes from tropical to temperate areas and consists of ca. 21 species in the globe^1^. Among these species, *O. alismoides* has been under extensive investigation. The species used to widely spread in China and presently under threat due to habitat fragmentation (Chen et al., 2008). As an annual species, the seed germination was featured with density dependence (Yin et al., 2009, 2013). In addition, *O. alismoides* was found with three carbon concentrating mechanisms, i.e., bicarbonate usage, Crassulacean acid metabolism (CAM) and C4 (Zhang et al., 2014; Shao et al., 2017), which showed potentially strong influence on carbon cycling in freshwater ecosystems dominated by the species (Maberly and Gontero, 2017; Shao et al., 2017). While other species in genus *Ottelia* were less investigated, and the main focus was about the phylogenetic relationship among these species based on the characteristic of isozyme, flower, seed and qualitative description of the species (Kaul, 1969; Cook et al., 1984; He, 1991). For instance, Chen et al. (2017) has studied five recorded varieties of *O. acuminata*, an endemic species in China, based on molecular proofs, and the authors stated that most of collected varieties reflected genetically differentiated group, and the genetic divergences could be linked with the past tectonic movements. Other than *O. alismoides*, the *Ottelia* species or varieties grew in a localized and relatively stable karst freshwater (Chen et al., 2017). Consistently, Li et al. (2018) assumed a fast speciation process of *O. acuminata* due to geographic features in these areas. As the dominant submerged species in pristine karst freshwaters, populations of *O. acuminata* are potentially important factors of carbon source/sink in the ecosystem (Wang et al., 2017). Therefore, an *in-situ* investigation of leaf traits could give the indispensable information of ecological functions of the *Ottelia* populations in the freshwater ecosystem.

In this study, we sampled eight *Ottelia* populations across the distance of 1,700 km in karst freshwaters in China, and we hypothesized that leaf traits of the *Ottelia* populations can correlate with phylogenetic relationship, and a detailed analysis of leaf traits facilitates to reveal the role of *Ottelia* populations in the carbon cycling in karst freshwaters.

## Materials and Methods

Eight populations in Luguhu (LG), Heqing (HQ), Jianchuan (JC), Eryuan (EY), Guiyang (GY), Songming (SM), Jingxi (JX), and Haikou (HN) were distributed in the provinces of Yunnan, Guizhou, Guangxi and Hainan, covering a distance of >1,700 km. Most of the collected species grew in localized karst freshwaters, and the geographic information of the sampling sites was listed in Table 1.

**Table 1:** The geographic information of the sampling sites.

Taxon	Population code	Location	Latitude (N)	Longitude (E)	Habitat
*O. acuminata* var. *crispa*	LG	Luguhu, Yunnan	27.67°	100.76°	Lake
*O. acuminata* var. *acuminata*	HQ	Heqing, Yunnan	26.55°	100.17°	Pond
	JC	Jianchuan, Yunnan	26.53°	99.96°	Pond and stream
	EY	Eryuan, Yunnan	26.16°	99.93°	Lake
*O. balansae*	GY	Guiyang, Guizhou	26.43°	106.67°	Pond
*O. acuminata* var. *songmingensis*	SM	Songming, Yunnan	25.27°	102.88°	Pond and stream
*O. acuminata* var. *jingxiensis*	JX	Jingxi, Guangxi	24.84°	103.45°	River
*O. cordata*	HN	Haikou, Hainan	19.94°	110.40°	Stream


The physico-chemical variables in each site including atmospheric pressure (AP), dissolved oxygen (DO), conductivity (C), total dissolved solid (TDS), pH, and oxidation–reduction potential (ORP) were measured *in situ* by a YSI Pro Plus multiparameter meter (Xylem, United States). Photosynthetically active radiation (PAR) was determined at the water depth of 0 cm and 40 cm by a LI-1400 Data Logger and a LI-192 underwater quantum sensor (LI-COR, United States), and the light attenuation was calculated assuming an exponential decay of PAR in the water column (Kirk, 1977). Two or more liters of water samples were collected by a plastic tube sampler and separated into aliquot for the determination of total nitrogen (TN), total phosphorus (TP), alkalinity (Alk), and phytoplankton chlorophyll a (PhyChla). Samples for TN and TP were frozen at -20°C, transferred into lab and determined by the K_2_S_2_O_8_ digestion (Huang et al., 1999). Alkalinity (Alk) was determined using the Gran titration of 0.1 M HCl. At least 1 l of water was filtered through the GF/C filter for the determination of PhyChla, and the filter was extracted by 95% ethanol and determined by a spectrophotometer (Jespersen and Christoffersen, 1987).

Based on Kraft et al. (2015), we used the combination of functional traits (not a single trait) to evaluate the ecological functions of the leaves of *Ottelia*. We classified the leaf traits into three categories: leaf morphology and mass, pigment content and parameters of chlorophyll fluorescence and carbon-related traits (bicarbonate usage, capability of CAM and photosynthetic characteristics).

The individuals of *Ottelia* were harvested gently from the ponds or rivers. Twenty intact leaves were randomly chosen for the determination of leaf morphology and fresh weight. The maximum width and the maximum length were measured by a ruler, and the thickness was determined by a vernier caliper. The leaf was then placed into a standard plate and photographed for the determination of the leaf area (Shao et al., 2017). Afterward, the leaf was blotted by an absorbent paper and submerged into a half-filled measuring cylinder, and the volume changes of the water in the cylinder was estimated as the leaf volume.

Leaf pigment content including chlorophyll a (Chla), chlorophyll b (Chlb), and carotenoids (Car) was measured by the extraction of 95% ethanol (Huang et al., 1999). The parameters of leaf chlorophyll fluorescence were determined by chlorophyll fluorometer MONITORING-PAM (Walz, Germany) following the methods in Kramer et al. (2004) and Jiang et al. (2017). Φ_PSII_ is derived from a pure ‘lake’ or ‘puddle’ model referring to the fraction of the energy absorbed by photosystem (PS) II that is used in photochemistry. Φ_NPQ_ and Φ_NO_ are used to estimate the flux of excitation energy into the non-photochemical pathways, and Φ_NPQ_ refers to the yield induced by downregulatory processes, Φ_NO_ for the yield of other energy losses. Φ_PSII_, Φ_NPQ_, and Φ_NO_ were obtained by an induction curve by setting the PAR of the active light at 127 μmol E^-1^ s^-2^. rETR_max_ and *I*_k_ were acquired by a rapid light curve using the 12 steps of PAR gradients from ca. 2 to 1500 μmol E^-1^ s^-2^.

The end-point pH in 1 mM Na/KHCO_3_ solution for the leaves of each population was determined by a pH-drift method (Zhang et al., 2014). CAM capability was determined according to the diel change of acidity in leaves using the titration of 0.01M NaOH to pH 8.3 (Shao et al., 2017). Photosynthesis rates were determined by measuring the changes of DO in the 50 ml glass bottles prior to and 30 min after adding the leaves into the bottle at the concentrations of 0, 1, 2, 4, 6, 8, and 10 mM dissolved inorganic carbon (DIC, represented as Na/KHCO_3_), respectively. As a commonly used method, the DIC in the water was stable during the determination (Zhang et al., 2014; Clement et al., 2016). The light was provided by LED light bulb (ca. 100 μmol E^-1^ s^-2^), and the dark respiration rates were determined in the brown bottles prior to and 30 min after adding the leaves into the bottle at the concentrations of 0 and 10 mM DIC. The net photosynthesis rates at different concentrations of DIC was fitted to a slightly modified Michaelis–Menten equation that considered the compensation point for DIC (Clement et al., 2016). The equation is:

$$\mathrm{Net}\;\mathrm{photosynthesis}\;\mathrm{rate}=\frac{V_{\max}*(\mathrm{DIC}-\mathrm{CP})}{K_{half}+(\mathrm{DIC}-\mathrm{CP})}$$

where (rate as mg O_2_ g^-1^ FW h^-1^ and concentration as mM) *V*_max_ is the maximum rate of net photosynthesis; CP is the DIC compensation concentration; *K*_half_ is the concentration of DIC producing half-maximal rates of net photosynthesis.

Five replicates were measured for the CAM capability, and three replicates were measured for other indicators of macrophyte leaves.

### Statistical Analysis

For most indicators of leaf traits, one-way ANOVA was used to analyze the difference among the eight populations. *Post hoc* test was conducted using Tukey method at the significance level of 0.05. The data was log-transformed to achieve variance homogeneity prior to the statistical analyses, if needed. Because the diel change of leaf acidity was determined by the difference of mean leaf acidity at dusk and at dawn and thus had no replicates, it was not quantitatively analyzed by ANOVA. *V*_max_ and *K*_half_ were estimated by fitting the Michaelis–Menten equation with standard errors (as stated above). The traits were classified into three categories: leaf morphology and mass, pigment content and parameters of chlorophyll fluorescence and carbon-related traits (bicarbonate usage, capability of CAM and photosynthetic characteristics). Pearson correlation and the cluster analysis were analyzed within each aspect. In this study the correlation coefficient that is >0.9 was defined as a strong relationship between the leaf traits, and only one trait was included in the further clustering analysis to reduce the statistical bias. The clustering analysis was conducted by package ‘mclust’ using the ‘euclidean distance’ after ‘scale’ the data. All the statistical analyses were determined in R 3.4.0. Data is presented in mean ± SD if not explicitly stated.

## Results

### Physic-Chemical Conditions in the Eight Sampling Sites

As shown in Table 2, most sites had low nutrient levels, low phytoplankton biomass, low light attenuation, and slight alkaline with relative high alkalinity, indicating a pristine status with clear water.

**Table 2:** The physic-chemical variables of the sampling sites.

Sampling sites	Temp (°C)	AP mmHg	DO mg L^-1^	C us cm^-1^	TDS mg L^-1^	pH	ORP mV	Kd m^-1^	Alk mmol L^-1^	PhyChla mg L^-1^	TN mg L^-1^	TP μg L^-1^
LG	21.1	550.9	6.63	219.2	153.4	8.94	-0.3	0.13	1.81	1.0	0.28	37
HQ	17.1	585.1	8.59	216.4	165.1	8.84	17.4	0.16	2.36	0.9	0.67	26
JC	16.3	584.8	14.7	288.5	213.6	7.98	167.2	0.58	3.15	2.5	0.69	29
EY	23.4	566.5	6.33	213.0	143.2	8.87	-27	0.72	1.43	5.0	1.21	59
GY	23.1	666.9	7.65	469.6	317.2	7.74	183.9	0.47	3.10	1.5	2.47	11
SM	20.3	607.9	7.25	281.5	204.1	8.39	114.6	—^a^	2.80	0.8	1.77	22
JX	22.2	685.8	6.52	430.6	297.3	7.79	51.2	0.31	3.95	0.5	2.31	29
HN	30.8	755.5	2.83	273.7	165.8	7.33	137.3	0.88	1.52	0.3	2.62	69


^*a*^*K*_*d*_ is not determined in the site SM.

### pH-Drift

**FIGURE 1:**
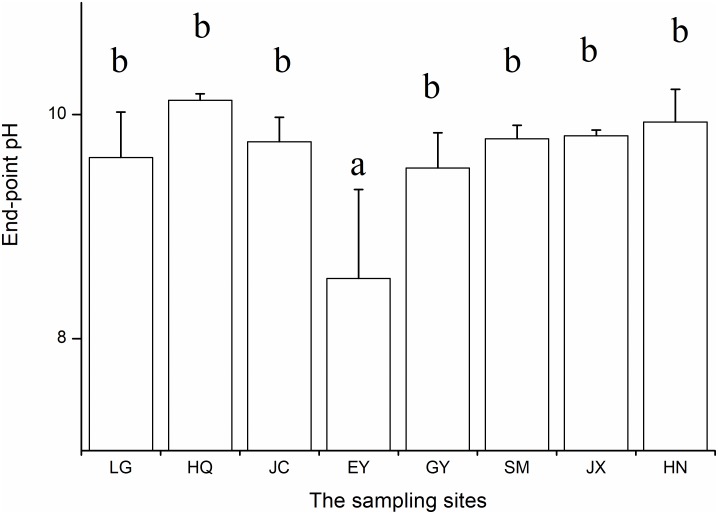
The end-point pH of the pH-drift experiments in the eight populations.

At the end of the pH-drift experiment pH was lowest in the EY population (Figure 1). After excluding the EY population, the end-point pH did not differ among the rest seven populations (ANOVA, *F* = 1.98, *p* > 0.05). Only the end-point pH in the HQ population is >10 (10.13 ± 0.06). Consistently, we observed that CaCO_3_ precipitation on the surface of the mature leaves in all eight populations, especially on two sides of leaves of *O. acuminata* var. *songmingensis.*

### Carbon-Related Leaf Traits

**FIGURE 2:**
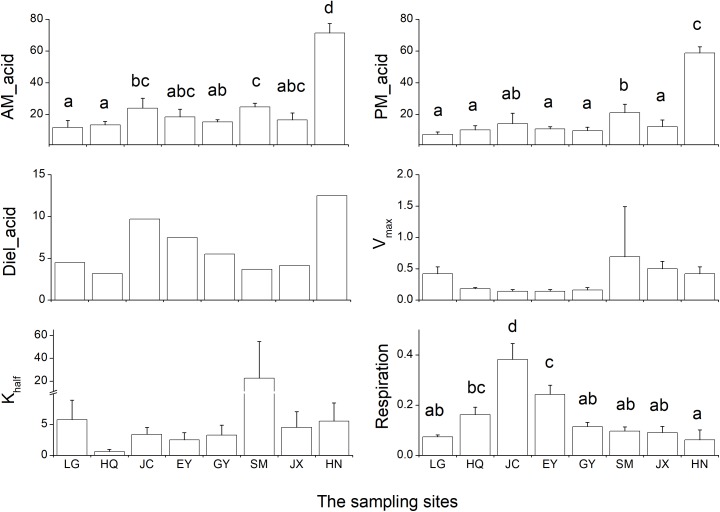
The six carbon-related leaf traits in the eight *Ottelia* populations. AM_acid and PM_acid is the acidity of the leaves at ca. 7 am and 7 pm (unit: μ equiv g^-1^ FW). Diel_acid is the changes between mean AM_acid and mean PM_acid (unit: μ equiv g^-1^ FW). *V*_max_ is the maximum rate of net photosynthesis (unit: mg O_2_ g^-1^ FW h^-1^), and *K*_half_ is the concentration of dissolved inorganic carbon producing half-maximal rates of net photosynthesis calculated from the Michaelis–Menten equation (unit: mM). Respiration is the respiration rate in the dark (unit: mg O_2_ g^-1^ FW h^-1^). Due to the method of calculation, Diel_acid, *V*_max_ and *K*_half_ are not analyzed by ANOVA and therefore no statistical results for *post hoc* test. The different letters in the figure indicate the significant difference among the eight population.

**FIGURE 3:**
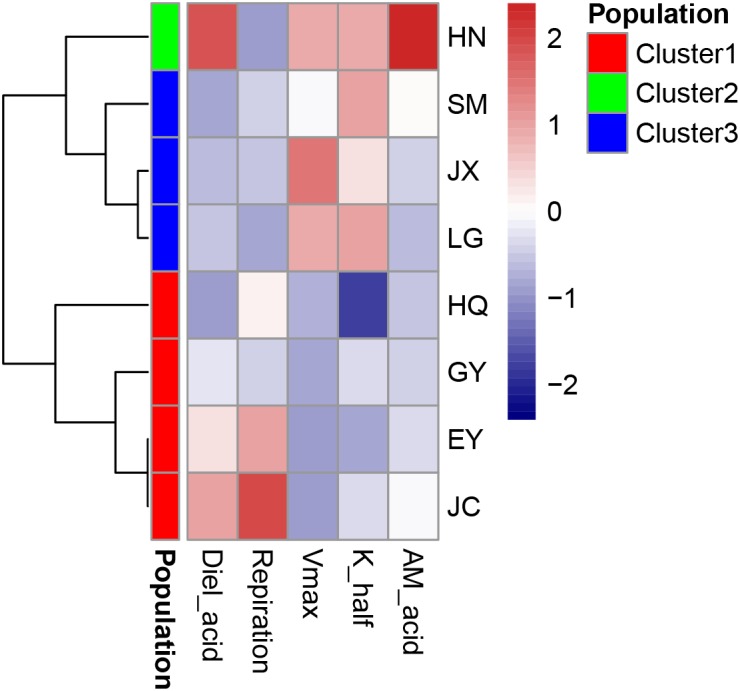
The cluster analysis of carbon-related leaf traits in the eight *Ottelia* populations. AM_acid is the acidity of the leaves at ca. 7 am (unit: μ equiv g^-1^ FW). Diel_acid is the changes of leaf acidity between ca. 7 am (dusk) and 7 pm (dawn) (unit: μ equiv g^-1^ FW). *V*_max_ is the maximum rate of net photosynthesis (unit: mg O_2_ g^-1^ FW h^-1^), and *K*_half_ is the concentration of dissolved inorganic carbon producing half-maximal rates of net photosynthesis calculated from the Michaelis–Menten equation (unit: mM). Respiration is the respiration rate in the dark (unit: mg O_2_ g^-1^ FW h^-1^).

The leaf acidity at dawn and dusk was highest in the HN population (Figure 2 and Supplementary Table S1). The largest diel acidity change was also detected in the HN population, arriving at 12.5 μequiv g^-1^ FW. *V*_max_ and *K*_half_ were both high in the SM population but with large variation among the replicates. The respiration rate in the dark was highest in the JC population, intermediate in the EY population, and lowest in the HN population. Since there was strong correlation between the leaf acidity at dusk and at dawn (Table 3), the further cluster analysis only included the leaf acidity at dusk. The hierarchical clustering revealed three clusters in the eight populations (Figure 3). The HQ, GY, EY, and JC populations were grouped into one cluster, and the HN population were one cluster, with the rest three as one cluster.

**Table 3:** The Pearson correlation between six carbon-related leaf traits in the eight populations.

	AM_acid	PM_acid	*V*_max_	Respiration	*K*_half_
Diel_acid	0.804	0.729	-0.027	0.240	0.182
AM_acid		0.993^∗^	0.307	-0.251	0.393
PM_acid			0.358	-0.336	0.416
*V*_max_				-0.718	0.693
Respiration					-0.525


*AM_acid and PM_acid is the acidity of the leaves at ca. 7 am and 7 pm (unit: μ equiv g^-*1*^ FW). Diel_acid is the changes between mean AM_acid and mean PM_acid (unit: μ equiv g^-*1*^ FW). *V*_*max*_ is the maximum rate of net photosynthesis (unit: mg O_*2*_ g^-*1*^ FW h^-*1*^). Respiration is the respiration rate in the dark (unit: mg O_*2*_ g^-*1*^ FW h^-*1*^). *K*_*half*_ is the concentration of dissolved inorganic carbon producing half-maximal rates of net photosynthesis calculated from the Michaelis–Menten equation (unit: mM). The star (^∗^) indicates a correlation coefficient > 0.9.*

### Leaf Pigment Content and Chlorophyll Fluorescence

**FIGURE 4:**
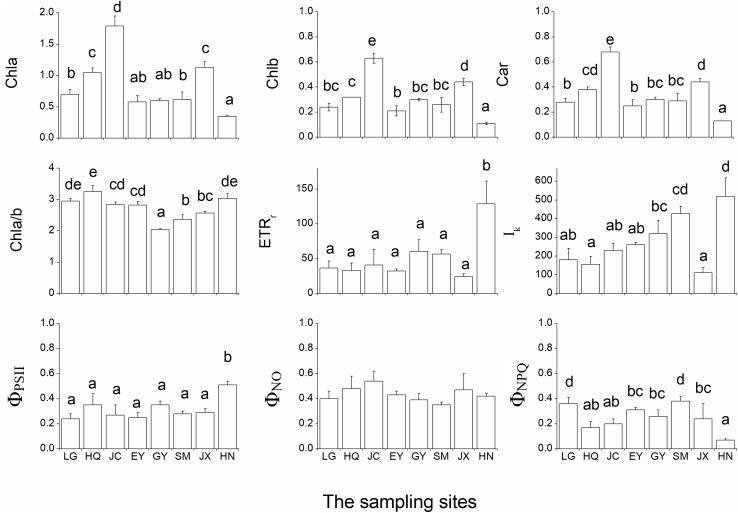
The nine pigment-related leaf traits in the eight populations. Chla, Chlb, Car, and Chla/b refer to the leaf chlorophyll a (unit: mg g^-1^ FW), chlorophyll b (unit: mg g^-1^ FW), carotenoids (unit: mg g^-1^ FW) and the ratio of Chla and Chlb, respectively. rETR_max_ (unit: μmol electron m^-2^ s^-1^), *I*_k_ (μmol photon m^-*2*^ s^-1^)_,_ Φ_PSII,_ Φ_NPQ_ and Φ_NO_ are five leaf chlorophyll fluorescence parameters in the inductive curve and rapid light curve. The different letters in the figure indicate the significant difference among the eight population.

The leaf chlorophyll a (Chla), chlorophyll b (Chlb) and carotenoids were highest in the JC population, intermediate in the JX population and lowest in the HN population (Figure 4). In contrast, the ratio of Chla and Chlb (Chla/b) was lowest in the GY population.

Both rETR_max_ and Φ_PSII_ were significantly higher in the HN than in the other populations (Figure 4). *I*_k_ was low in the HQ and JX populations and high in the SM and HN populations. Φ_NO_ did not differ among the eight populations while Φ_NPQ_ was highest in LG and SM populations.

**FIGURE 5:**
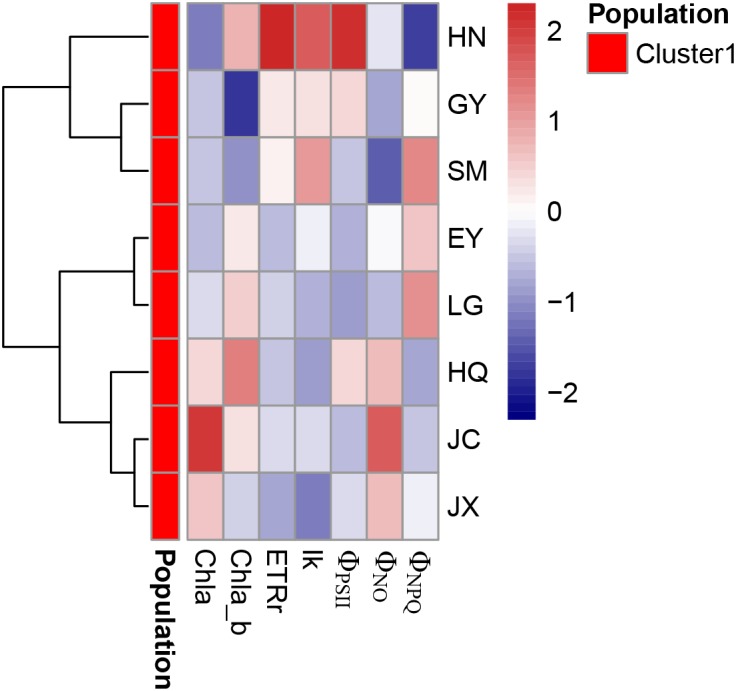
The cluster analysis of pigment-related leaf traits in the eight populations. Chla, Chlb, Car, and Chla/b refer to the leaf chlorophyll a (unit: mg g^-1^ FW), chlorophyll b (unit: mg g^-1^ FW), carotenoids (unit: mg g^-1^ FW) and the ratio of Chla and Chlb, respectively. rETR_max_ (unit: μmol electron m^-2^ s^-1^), *I*_k_ (μmol photon m^-2^ s^-1^)_,_ Φ_PSII_, Φ_NPQ_, and Φ_NO_ are five leaf chlorophyll fluorescence parameters in the inductive curve and rapid light curve.

The strong correlation was found among Chla, Chlb, and carotenoids (Table 4). The cluster analysis discovers only one cluster for the eight populations (Figure 5).

**Table 4:** The Pearson correlation between nine pigment-related leaf traits in the eight populations.

	Chlb	Chla/b	Car	rETR_max_	*I*_k_	Φ_PSII_	Φ_NO_	Φ_NPQ_
Chla	0.968^∗^	0.167	0.987*	-0.524	-0.583	-0.415	0.833	-0.117
Chlb		-0.083	0.991*	-0.528	-0.542	-0.448	0.732	-0.031
Chla/b			0.016	0.047	-0.188	0.180	0.474	-0.426
Car				-0.556	-0.563	-0.475	0.761	-0.024
rETR_max_					0.880	0.872	-0.311	0.576
I_k_						0.62	-0.534	-0.231
Φ_PSII_							-0.075	-0.822
Φ_NO_								-0.507


*Chla, Chlb, Car, and Chla/b refer to the leaf chlorophyll a (unit: mg g^-*1*^ FW), chlorophyll b (unit: mg g^-*1*^ FW), carotenoids (unit: mg g^-*1*^ FW) and the ratio of Chla and Chlb, respectively. rETR_*max*_ (unit: μmol electron m^-*2*^ s^-*1*^), *I*_*k*_ (μmol photon m^-*2*^ s^-*1*^)_,_ Φ_*PSII*,_ Φ_*NPQ*_ and Φ_*NO*_ are five leaf chlorophyll fluorescence parameters in the inductive curve and rapid light curve. The star (^∗^) indicates a correlation coefficient > 0.9.*

### Leaf Traits of Morphology and Mass

**FIGURE 6:**
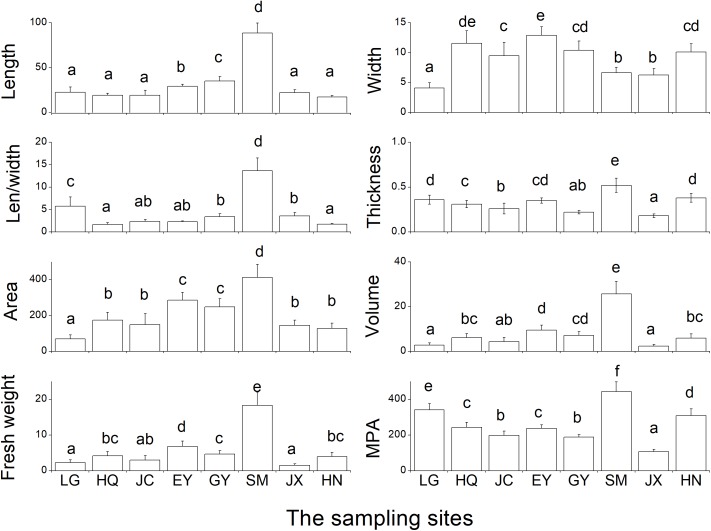
The eight leaf traits of morphology and biomass in the eight populations. Len/width refers to the ratio of leaf length and leaf width. MPA refers to the biomass per area of the determined leaf (unit: g m^-*2*^). Leaf length and width is with the unit of cm, thickness with the unit of mm, area with the unit of cm^*2*^, and volume with the unit of cm^*3*^, fresh weight with the unit of g. The different letters in the figure indicate the significant difference among the eight population.

The SM population had the largest leaf length (reaching ca. 100 cm), length/width ratio (Len/width), thickness, area, volume, fresh weight, MPA compared with the rest seven populations (Figure 6). While for leaf width, the EY population had the highest values, the LG population the lowest.

**FIGURE 7:**
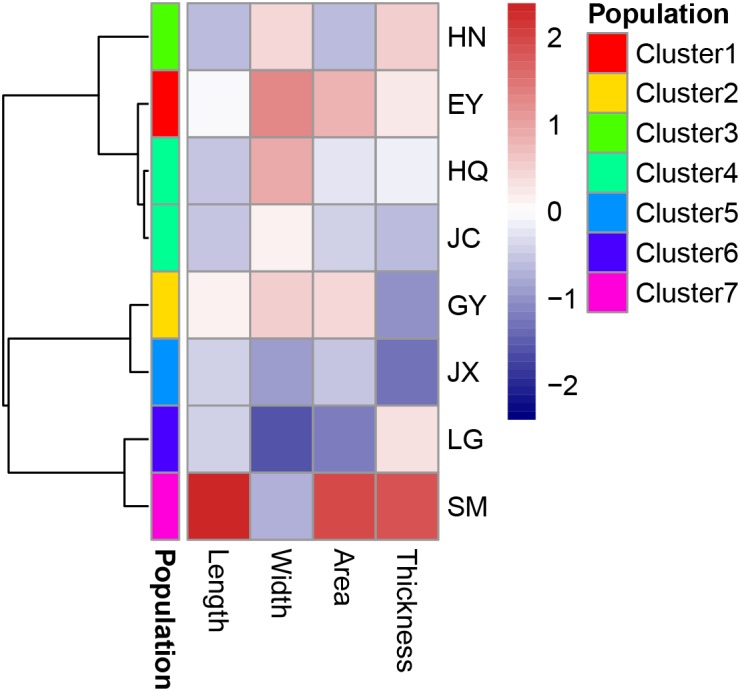
The cluster analysis of leaf traits of morphology and biomass in the eight populations. Leaf length and width is with the unit of cm, area with the unit of cm^2^ and thickness with the unit of mm.

There was strong correlation among several traits of leaf morphology and mass (Table 5), and only four traits (leaf length, width, area and thickness) were included in the further hierarchical clustering analysis (Figure 7). Seven clusters were identified with the HQ and JC populations as one cluster, and other populations are distinct from each other.

**Table 5:** The Pearson correlation between eight leaf traits of morphology and biomass in the eight populations.

	Width	Len/width	Area	Thickness	Volume	FW	MPA
Length	-0.243	0.933*	0.870	0.680	0.960*	0.961*	0.649
Width		-0.560	0.231	-0.151	-0.033	-0.051	-0.289
Len/width			0.646	0.694	0.840	0.850	0.717
Area				0.510	0.908*	0.901*	0.396
Thickness					0.780	0.795	0.964*
Volume						0.999*	0.706
FW							0.722


*Len/width refers to the ratio of leaf length and leaf width. FW refers to the fresh weight (unit: g). MPA refers to the biomass per area of the determined leaf (unit: g m^-*2*^). Leaf width is with the unit of cm, thickness with the unit of mm, area with the unit of cm^*2*^, and volume with the unit of cm^*3*^. The star (^∗^) indicates a correlation coefficient > 0.9.*

## Discussion

Based on 24 leaf traits, we have provided a quantitative profile of the eight *Ottelia* populations in field. After grouped into three aspects (traits of morphology and mass, carbon-related traits and leaf pigment and chlorophyll florescence parameters), the leaf traits showed different divergences in these populations. The physiological traits (such as diel acidity changes, leaf florescence parameters, and etc.) were usually measured in the studies of macrophytes exposed to toxicity or under other stress due to the sensitive responses (Zhang et al., 2014; Shao et al., 2017). While in this study, the physiological traits were less sensitively divergent than morphological ones (seven clusters) among the eight populations, probably reflecting the physiological adaptation to the clear water conditions benign for the growth of *Ottelia*. The quantitatively determined traits showed to some extent correlation with the phylogenetic relationship, implying that variation or speciation of *Ottelia* in these isolated water systems have produces various traits in each population. For example, the morphological traits were clustered into seven groups (the two populations from the same *O. acuminata* var. *acuminata* were merged into one cluster), and the HN population was singled out base on the carbon-related traits. This is consistent with the results of He (1991) using several qualitative or semi-quantitative traits such as flower, seed and other structures. We also found special features within two populations. The SM population (*O. acuminata* var. *songmingensis*) had thickest and longest leaves in all the population and interestingly with calcium precipitation on both sides of the leaves, an interesting phenomenon that requires further study. The HN population (*O. cordata*) had high diel acidity changes, only similar with *O. alismoides* but not with other species in the genus *Ottelia* in China, which provided a clue for future phylogenetic research.

The leaf functional traits have been well investigated in terrestrial plants, and the photosynthesis rate, leaf weight per area and leaf life span of >2,000 species of plants have been recorded (Wright et al., 2004). The authors proposed a LES and claimed that there was a typical trade-off between leaf functional traits that leaves with high photosynthesis rate are featured low life span and low leaf MPA (Wright et al., 2004). To our knowledge, this study is the first attempt to systematically analyze the leaf functional traits in submerged freshwater macrophytes. The low leaf MPA and high photosynthesis rate of the *Ottelia* leaves fit well with LES and placed in the spectrum of the quick return on nutrient and biomass investment. Accordingly, the leaf life span of *Ottelia* was expected to be short as other submerged species (Hemminga et al., 1999; Yamamoto et al., 1999; Kamermans et al., 2001). In addition, Cornwell et al. (2010) revealed the predominant influence of plant functional traits on decomposition rates at a global scale. Based on our findings on functional traits of *Ottelia* populations, the decomposition rate and carbon turnover of these populations should be very fast, similar to *Potamogeton crispus* (Wang et al., 2016), and thus a fast carbon cycling was expected for the *Ottelia*-dominated karst freshwaters. However, our results, together with Yin et al. (2017), indicated that all the species or varieties of the genus *Ottelia* in China could use bicarbonate as inorganic carbon supply though there was variation among the populations. Similar like *Potamogeton lucens* (Prins et al., 1982), the *Ottelia* leaves were expected with polarity of pH between the adaxial and abaxial side, but with much broader leaves probably stronger effects on the polarity. Therefore, the calcium precipitation (as CaCO_3_) on the surfaces of *Ottelia* leaves could bring abundant carbon burial when the macrophytes were dominant producers and thus strongly affect the carbon cycling. Thirdly, high diel acidity changes >10 μequiv g^-1^ FW were found even at high inorganic carbon supply, which is potentially inducible CAM feature similar with that *O. alismoides* showed in Shao et al. (2017). A dense macrophyte bed with strong CAM capacity could also affect the pH of water column at night (Keeley, 1983). Consequently, the changes of pH have a strong effect on the inorganic carbon species in the water column and other primary producers, e.g., periphyton on the leaves (Maberly, 1996; Hao et al., 2017). In summary, the role of *Ottelia* populations on the carbon cycles in the karst freshwaters warrants further studies.

There is also small variation of plant traits among the three populations (HQ, EY, and JC) belonging to the same variety *O. acuminata* var. *acuminata*. The lower end-point pH in EY population concurred with habitat fragmentation and destruction in Eryuan County. During our field survey, we found that the natural populations of *O. acuminata* var. *lunanen* and *O. emersa* disappeared due to habitat destruction. The extinct of the *Ottelia* populations should be highlighted in the future projects of macrophyte conservation.

## Conclusion

Our trait analyses profiled the eight populations of genus *Ottelia* in details. With the unique growth form Otteliids and limited distribution in the localized area, the investigated species or varieties may form special effects on the growing freshwaters distinct from other submerged species. Our results indicated an important ecological role of submerged macrophyte *Ottelia* spp. in the karst freshwaters. More studies about whether direct uptake of bicarbonate or relying on extracellular carbonic anhydrase in *Ottelia* leaves would provide more accurate knowledge about the effects of the species on other primary producers and the carbon cycling in the karst freshwaters.

## Author Contributions

YC, WL, and HJ designed the experiments. YC and LX determined the physic-chemical variables. YL determined the leave morphology. LN determined the photosynthesis rate. HJ determined the acidity of leaves. YC and HJ wrote the first edition of the manuscript, and other co-authors contributed to the modification of the manuscript.

## Conflict of Interest Statement

The authors declare that the research was conducted in the absence of any commercial or financial relationships that could be construed as a potential conflict of interest.

## Footnotes

1 http://foc.eflora.cn/content.aspx?TaxonId=123432, accessed on 06-28-2018.
