# Supplementary material for: The Analysis of Leaf Traits of Eight Ottelia Populations and Their Potential Ecosystem Functions in Karst Freshwaters in China
Source: Front Plant Sci. 2019 Jan 7;9:1938. doi: 10.3389/fpls.2018.01938 (PMC6330901; doi:10.3389/fpls.2018.01938)
Supplement: Supplementary file 1 [file Table_1.DOCX]

| Population | V_max_ | K_half_ | CP | R² |
| --- | --- | --- | --- | --- |
| LG | 0.42 ± 0.11 | 5.79 ± 3.14 | 0.23 ± 0.29 | 0.922 |
| HQ | 0.18 ± 0.02 | 0.61 ± 0.35 | ND | 0.515 |
| JC | 0.14 ± 0.03 | 3.39 ± 1.15 | 1.58 ± 0.35 | 0.749 |
| EY | 0.14 ± 0.03 | 2.49 ± 1.18 | 0.87 ± 0.3 | 0.724 |
| GY | 0.16 ± 0.04 | 3.25 ± 1.63 | 1.06 ± 0.34 | 0.786 |
| SM | 0.69 ± 0.80 | 22.5 ± 32.32 | 1.82 ± 0.49 | 0.989 |
| JX | 0.50 ± 0.12 | 4.54 ± 2.54 | ND | 0.9 |
| HN | 0.42 ± 0.11 | 5.52 ± 2.98 | ND | 0.71 |

Table S1. The photosynthetic traits of the leaves estimated from the different photosynthesis rates in different dissolved inorganic carbon (DIC) concentrations in the eight populations. V_max_ is the maximum rate of net photosynthesis; CP is the DIC compensation concentration; K_half_ is the concentration of DIC producing half-maximal rates of net photosynthesis. R^2^ indicates the fitness using the Michaelis-Menten equation. ND means that the best fitted model did not produce a reasonable estimation for CP and therefore CP is removed from the Michaelis-Menten equation.
